# eGFR slope as a primary endpoint for clinical trials of CKD progression: one size fits all?

**DOI:** 10.1093/ckj/sfae001

**Published:** 2024-01-04

**Authors:** Balazs Odler, Edouard L Fu

**Affiliations:** Division of Nephrology, Department of Internal Medicine, Medical University of Graz, Graz, Austria; Department of Clinical Epidemiology, Leiden University Medical Center, Leiden, The Netherlands

Traditionally, randomized controlled trials evaluating the efficacy of drugs in chronic kidney disease (CKD) have used clinical endpoints that represent late events in the disease course, i.e. kidney failure with replacement therapy (KFRT) or doubling of serum creatinine [1]. However, such outcomes require large sample sizes, long follow-up times and inclusion of patients with later stages of CKD for sufficient power. In addition, treatment effects are driven by the small subset of fast progressors that develop the clinical endpoint during the trial. Use of surrogate endpoints such as estimated glomerular filtration rate (eGFR) slope may overcome these limitations.

**Figure 1:**
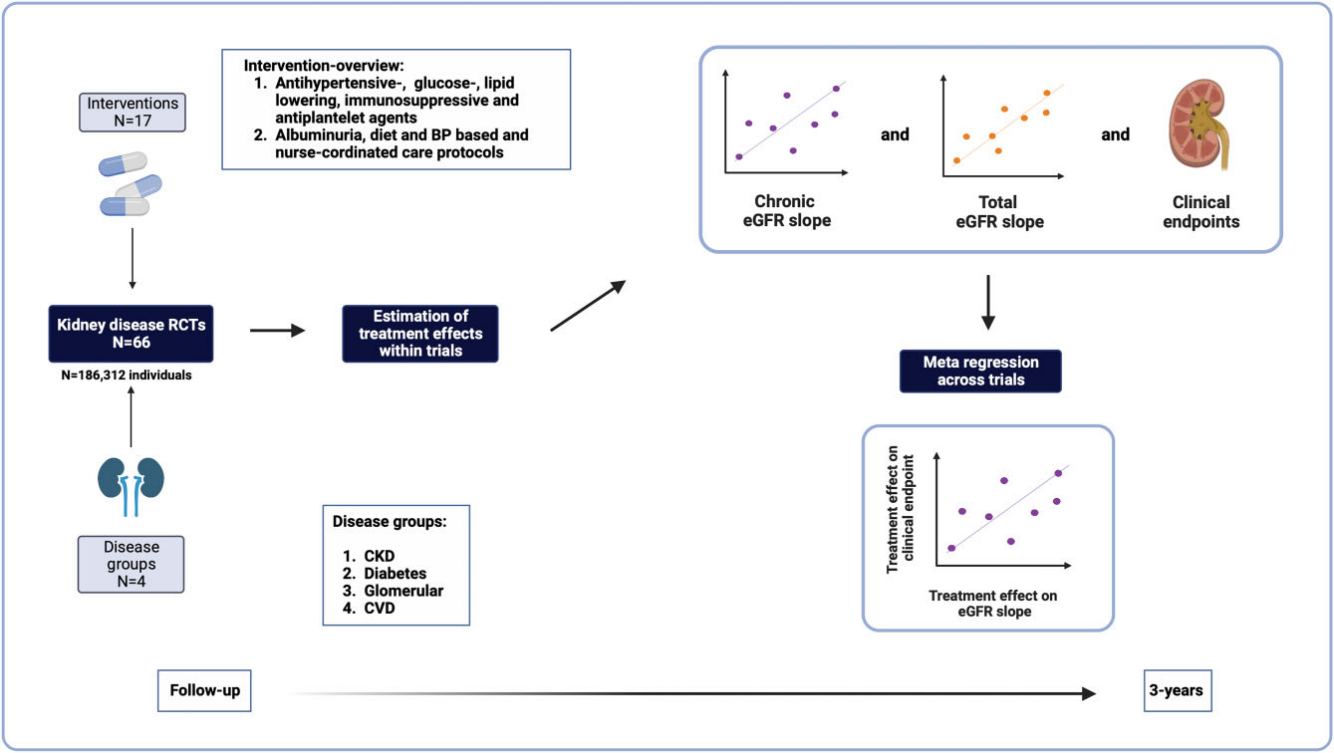
Overview of the meta-analysis.

The meta-analysis by Inker *et al*. investigates whether treatment effects on eGFR slope reliably predict treatment outcomes on kidney failure—a key criterion for valid surrogate outcomes [2] (Fig. 1). Specifically, the study included individual patient data of 186 312 participants from 66 randomized trials, which tested 17 interventions in four broad disease groups (diabetes, glomerular disease, CKD and cardiovascular disease). In comparison with an earlier analysis [3], the study population includes 19 more trials (including 7 cardiovascular trials) and over 125 000 additional participants. Within each trial, the authors estimated treatment effects on the 3-year total eGFR slope, chronic eGFR slope (calculated from Month 3 onwards) and the clinical endpoint, which was defined as a composite of KFRT (dialysis or kidney transplant), sustained GFR <15 mL/min/1.73 m^2^ and doubling of serum creatinine. Thereafter, Bayesian meta-regression was used across all trials to predict treatment effects on the clinical endpoint from treatment effects on the eGFR slope. Ideally, the meta-regression line has a large slope (indicating that treatment effects on eGFR slope strongly predict effects on the clinical endpoint) and an intercept of zero (indicating that if the treatment effect on the eGFR slope is zero, the treatment effect on the clinical endpoint is also null); furthermore, a large R^2^ indicates that most of the variation in treatment effect on the clinical endpoint is explained by variation in treatment effects on eGFR slope.

There was a strong association between treatment effects on total eGFR slope and treatment effects on the clinical endpoint, with an R^2^ of 0.97 and a meta-regression slope indicating that each 0.75 mL/min/1.73 m^2^/year greater benefit on total eGFR slope was associated with a 23.3% lower hazard ratio for the clinical endpoint. Different disease groups, severity of kidney disease and exclusion of doubling serum creatinine from the clinical endpoint had no major influence on results for total slope, although the intercept was significantly different from 0 when using 2-year total eGFR slope. R^2^ was 0.55 when using chronic eGFR. However, the meta-regression slope had a similar magnitude to that of total eGFR and its intercept was close to zero.

Validated surrogate endpoints reflecting early changes in the disease course for clinical trials of CKD progression are particularly needed. This meta-analysis showed good performance of 3-year total eGFR slope and supports its use as a surrogate endpoint for clinical trials in CKD. Nevertheless, choices on duration of follow-up and total vs chronic slope will need to be made on a case-by-case basis, taking into account trial design, disease area and specific intervention studied.
